# Experience Changes How Emotion in Music Is Judged: Evidence from Children Listening with Bilateral Cochlear Implants, Bimodal Devices, and Normal Hearing

**DOI:** 10.1371/journal.pone.0136685

**Published:** 2015-08-28

**Authors:** Sara Giannantonio, Melissa J. Polonenko, Blake C. Papsin, Gaetano Paludetti, Karen A. Gordon

**Affiliations:** 1 Department of Head and Neck Surgery, Institute of Otorhinolaryngology, Catholic University of the Sacred Heart, Rome, Italy; 2 Archie’s, Cochlear Implant Laboratory, Department of Otolaryngology, The Hospital for Sick Children, Toronto, ON, Canada; 3 Institute of Medical Sciences, The University of Toronto, Toronto, ON, Canada; 4 Department of Otolaryngology—Head and Neck Surgery, University of Toronto, Toronto, ON, Canada; Max Planck Institute for Human Cognitive and Brain Sciences, GERMANY

## Abstract

Children using unilateral cochlear implants abnormally rely on tempo rather than mode cues to distinguish whether a musical piece is happy or sad. This led us to question how this judgment is affected by the type of experience in early auditory development. We hypothesized that judgments of the emotional content of music would vary by the type and duration of access to sound in early life due to deafness, altered perception of musical cues through new ways of using auditory prostheses bilaterally, and formal music training during childhood. Seventy-five participants completed the Montreal Emotion Identification Test. Thirty-three had normal hearing (aged 6.6 to 40.0 years) and 42 children had hearing loss and used bilateral auditory prostheses (31 bilaterally implanted and 11 unilaterally implanted with contralateral hearing aid use). Reaction time and accuracy were measured. Accurate judgment of emotion in music was achieved across ages and musical experience. Musical training accentuated the reliance on mode cues which developed with age in the normal hearing group. Degrading pitch cues through cochlear implant-mediated hearing induced greater reliance on tempo cues, but mode cues grew in salience when at least partial acoustic information was available through some residual hearing in the contralateral ear. Finally, when pitch cues were experimentally distorted to represent cochlear implant hearing, individuals with normal hearing (including those with musical training) switched to an abnormal dependence on tempo cues. The data indicate that, in a western culture, access to acoustic hearing in early life promotes a preference for mode rather than tempo cues which is enhanced by musical training. The challenge to these preferred strategies during cochlear implant hearing (simulated and real), regardless of musical training, suggests that access to pitch cues for children with hearing loss must be improved by preservation of residual hearing and improvements in cochlear implant technology.

## Introduction

Basic components of music (mode and tempo) influence our perception of whether the music is happy or sad [1, 2]. In the present study, we examined how developmental experience shapes the use of these cues. Our specific aims were to identify effects of: 1) musical training during typical childhood; 2) reduced access to mode cues during development in children who are deaf and use bilateral cochlear implants (CI) or a cochlear implant in one ear and hearing aid in the other to hear and 3) compensation for acute reduction of mode cues.

### A) Distinct components of music are processed in the brain to convey emotions

Like speech, music is used in a unique way by humans to communicate ideas and emotions [3]. Since both speech and music appear early in human development, a neural architecture has been hypothesized to explain how these capacities emerge [4]. In particular, music requires a nervous system that is able to encode its basic elements with great accuracy, possibly to a greater extent than speech. Music’s main features include pitch and rhythm, which appear to be processed independently by separate neural subsystems, as identified in brain damage studies [5, 6]. These elements are organized in a hierarchy that reflects the specialization of specific brain areas: tonal hierarchy (pitch relations in scales) develops early and is computed in the right temporal neocortex and posterior secondary auditory cortex [6], while harmonic hierarchy (pitch relations in chords) appears later in development [7, 8] and is processed bilaterally in the frontal operculum [9]. Rhythmic grouping (segmentation into temporal groups of event) is processed in the left temporal auditory cortex, whereas rhythmic regularity (meter, or underlying beat) is functionally dissociated in the right temporal auditory cortex and lateral cerebellar hemisphere [10, 11]. Music elicits pleasure by activating dopamine-dependent reward/reinforcement systems (including dorsal midbrain, ventral striatum, insula and orbitofrontal cortex activation) via inputs from neocortex [12].

Humans are known to be the only species able to obtain pleasure from aesthetically abstract or conceptually meaningful stimuli (such as listening to music), with little direct relevance for survival [13]. It is widely believed that the pleasure people experience from music is related to emotions induced by music itself [4]. Associations between specific music characteristics and emotions are well established [14, 15], especially for happiness and sadness [1, 2]. Such associations are evident across musical cultures [16]. Mode and tempo are considered to be the key elements to induce these two basic emotions [17]. Major chords (Ionian Western diatonic scale: four semitones between the first and third pitch of a given scale) and faster tempi (many beats per minute) convey happiness, whereas minor chords (Aeolian Western diatonic scale: three semitones between the first and third pitch of a given scale) and slower tempi (few beats per minute) are known to induce sadness in the listener [2, 14–20].

There is a lack of systematic investigation of emotional processing of music in the classic literature, for it was thought to be a very personal and variable experience. More recently, however, various mechanisms have been advocated to explain how music listening may induce emotions, including brain stem reflexes, evaluative conditioning, emotional contagion, visual imagery, episodic memory, and musical expectancy [21]. Emotional interpretation of music relies on specific musical properties with consistent processing across individuals and age. The emotional valence in music can be identified within one second [22], or even as little as half second [17], and children as young as three years old are able to discriminate happy from sad tunes [2, 20].

While tempo perception seems to be more intuitive, immediate and independent from the musical cultural context in which it develops, the discrimination between major and minor chords is based on implicit knowledge or passive exposure to pitch structures of Western music [7]. Tempo offers an earlier access to emotion processing than mode, whose sensitivity emerges later in life [23]. Whereas young children rely primarily on tempo rather than mode to judge emotion in music [23, 24], adults use a complex combination of tempo, loudness, pitch level, mode, and consonance/dissonance [16] though both mode and tempo remain the most relevant factors [17].

The saliency of tempo cues in early life could be explained by the assumption that tempo is easier to process or that sensitivity to mode is more dependent on music learning with age [25]. Unfortunately, little is known about the specific and relative contribution of mode and tempo across the life span, how it varies and how it is influenced by musical exposure from childhood to adulthood. In Experiment 1, we aimed to determine the effects of age and specific training on normal development of musical emotion identification skills, and to examine how these variables affect the strategy used to recognize emotions in musical excerpts.

### B) Emotion perception in music is likely shaped by experience during development

Brain anatomy can change noticeably as a function of learning, both in adults and children [26, 27]. Formal musical training can be a multimodal experience involving the visual, auditory and somatosensory modalities as well as the motor system [28], and represents a unique model in which to study plastic changes in the human brain [29–31]. Experience can shape, reorganize and enhance a wide range of brain regions [32–34], both structurally in terms of cytoarchitecture (i.e., greater volume of tissue) [3–36] and functionally in terms of connectivity (i.e., different neural recruitment, synchronized firing pattern) [37]. Music training affects vascularization, synaptogenesis, glial cells, and increases gray-matter volume, cortical thickness and reorganizes white matter [35, 38]. Evoked cortical P2 wave components are larger in skilled musicians [39], and are highly neuroplastic with both pitch-based [40] and rhythm-based discrimination training [28]. Larger amplitude responses from auditory cortex have been observed after sensorimotor-auditory training rather than after auditory-only training, suggesting that multimodal stimulation has a greater effect on auditory cortex than auditory stimulation alone [41]. Musically trained adults [42] and children [43] have significantly larger electrophysiological measures of pre-attentive discrimination between major and minor chords than controls (mismatched negativity response) particularly above the right hemisphere, consistent with the theory of right cortical hemispheric dominance for spectral processing proposed by Zatorre and Belin [44].

The degree of anatomical and functional change induced by musical experience is related to the number of years of continuous musical training [45], amount of practice [46], age of formal training onset [47, 48], and possibly aptitude [49]. Predisposition related effects, which coexist and interact with training, could explain the heterogeneity of training outcomes. Slower and faster learners may be categorized as a function of fine-grained encoding of pitch information or levels of functional connectivity in the auditory cortex [50, 51]. The source of individual differences remains unknown, but is likely to rely on interactions between genetic/epigenetic mechanisms with environmental factors. Some authors advocate for an age range (6–8 years) of an alleged “musical sensitive period”, after which the effect of age at initiation of musical training decreases or plateaus [52, 53].

The scientific literature agrees about the positive and negative connotations conveyed by specific aspects of music such as tempo and mode, but to date only a few studies have investigated infants’ emotional response to music and how emotional associations to musical characteristics develop over time. Infants as young as 7 to 11 months are able to detect mode changes [54] but only few studies focused on children’s sensitivity to mode itself, which appears present in 8 year old children but not in 7 year olds [55].

This led to the idea that the ability to attribute emotional connotation based on mode cues alone develops with cognitive maturity and exposure to the musical culture. Gerardi and Gerker described a “two-step” developmental change, in which 5 year olds’ discrimination of emotion in music based on mode clusters near chance, while by age 8 years there is a shift towards more positive affect in response to major melodies, with the greatest differentiation between modalities found in adulthood. The authors therefore hypothesized the existence of a developing sensitivity to tonal scale structure that facilitates or underlies modal differentiation [25, 56].

Taken together, the ability to assign mode an emotional connotation takes time to develop, for it requires sufficient musical exposure and cognitive maturity [57]. These questions were explored in Experiment 1. We hypothesized that there would be an interaction between improvements in emotion identification with age and music exposure, reflecting the combined importance of both time and access to music in children with normal hearing.

### C) Perception of music is altered by cochlear implant hearing

Given the importance of experience to shape music perception during development, it is likely that children wearing cochlear implants use unique strategies to make judgments about the emotion conveyed in music. Indeed, access to the normally rich soundscape through a CI is restricted by its signal processing limits, which are responsible for poor pitch resolution and atypical timbre quality [58]. In Experiment 2 we tested which components of music children using CIs used to judge emotions in music. Hearing abilities of children with bilateral CIs were compared with children using a CI in one ear and a hearing aid in the other non-implanted ear (bimodal users) and age-matched typically developing children with normal hearing.

Many causes are proposed to explain poor music perception in CI users [59–61] including: 1) the limited number of active channels (8–22 physical contacts with the ~30,000 human acoustic nerve fibers), and 2) coding strategies providing only a rough estimate of spectral shape and little of the fine-grained temporal structure in sound [58]. Although children have been able to develop age equivalent speech and language abilities using this unique input, the poor representation of sound has negatively impacted music perception for both adults [58, 62] and children [63–66]. CI users have similar accuracy in rhythm based tasks compared to people with normal hearing [67, 68], whereas poor pitch perception in CI users negatively affects identification of instrument [69], pitch [70] and familiar melodies [71].

Musical experience of child CI users has generally been limited to CI input because their deafness was from birth or very early in life. As a result, they do not compare the quality of sound through their CI(s) with a remembered sound as do adults with post-lingual deafness and they seem to enjoy and take part in musical activities to the same extent as their hearing peers [72, 73]. Children with congenital or early onset deafness lack a mental representation for what constitutes normal pitch relations among notes, which could be perceived as flat, compressed, distorted or even reversed [58], but “normal and natural”, nonetheless. Considerable intra- and inter-subject variability exists due to hearing history, life experience and developmental level in functional areas that support musical skills, such as cognition and motor skills, which could explain the great heterogeneity of the results [60, 74].

Thus, given the technical constraints of signal transduction, which places a greater importance to the rate of stimulation rather than a faithful reproduction of the envelope of the waveforms, unilateral CI users rely more on tempo than mode cues because of the signal from the CI device itself [73, 75]. If this holds true for children using unilateral CIs, it may or may not be true of children using bilateral CIs. Recently, due to the growing body of literature, which demonstrated the importance of binaural hearing, many unilaterally implanted patients have received a second implant at a variable length of time from the first CI (sequential bilateral implantation). In such case, there may be a mismatch in the information sent through the auditory system by the two ears, either because of asymmetric development/experience occurring from sequential implantation [76] or because of abnormal binaural hearing [77, 78]. Sequential implantation with delays exceeding 1.5 years result in mismatched activity along the bilateral auditory brainstem [79] and cortex [76]. Although children with bilateral CIs are able to perceive binaural cues [77], children receiving bilateral CIs sequentially retain a preference for their first implanted ear and may often hear the input from both devices separately rather than one fused image [78]. The impact of bilateral CI use in children on music perception remains a question and is thus explored in Experiment 2.

Thanks to broadening inclusion criteria for cochlear implantation, today there are many children receiving one implant who have considerable residual hearing in the contralateral ear. These children can therefore still benefit from hearing through a conventional hearing aid in one ear and a cochlear implant in the other ear (bimodal hearing). This emerging population has a theoretical advantage for music perception over children using unilateral or bilateral CIs because of their access to low-frequency acoustic information. A group of children using bimodal devices were included in Experiment 2 to explore whether the combined use of acoustic and electric hearing by children is of benefit for identifying emotion in music.

Similar to individuals with normal hearing, training is a powerful tool proven to improve music identification and appreciation in CI users. Perceptual accuracy in pitch- and rhythm-based tasks, as well as music enjoyment, can be enhanced in CI recipients by specific music-oriented training programs [74]. Yet it is unclear whether musical training would change children’s preferred use of tempo or mode cues to identify the affective content of musical excerpts.

We hypothesized that CI and bimodal users would show poorer accuracy than normal hearing peers in identifying emotions conveyed by the original musical excerpts. Moreover, we hypothesized that bilateral CI users would use tempo more than mode cues when asked to identify the emotion in musical excerpts. Bimodal users would have a relatively greater reliance on mode cues than CI users due to their greater access to acoustic information. These findings would be independent of any formal musical training.

### D) Adaptation to abnormal input: a developmental change or quick adjustment?

In Experiment 3, we asked whether any differences in music emotion identification in children with CIs reflect developmental changes or, rather, more rapid abilities to adjust to changes in stimuli. We tested this by comparing responses of CI users with responses of children with normal hearing when mode cues were degraded by vocoded processing.

While abnormal input induces plasticity in the auditory system, the normal system is able to adapt to changing acoustic environments as well. Contributing mechanisms are fast “ad-hoc” adaptive processes as well as longer term changes. Plasticity is constantly occurring in the auditory system; rapid changes in early development take place at all levels of the auditory system (e.g., endbulb of Held [80]; brainstem [81–84]; cortex [76, 85], and post-synaptic cell potentials in the auditory system are continuously modified with experience, background activity and rapid changes in stimuli [86]. Slower adaptation to sound, such as adjusting to an unfamiliar accent, persists over long periods of time [87]. Short-term modulations ultimately lead to long-term plasticity, which in turn supports perceptual learning, along with auditory sensory memory, sensory predictions, novelty detection, selective attention and audiovisual integration [36, 86]. It is therefore possible that the auditory system in individuals with normal hearing could adapt to impoverished acoustic input even in an acute/short-term situation. We hypothesized that, like child CI users, children with normal hearing would increasingly rely on tempo cues to judge the emotion in music as mode cues were degraded and that their accuracy as determined by the test would fall to levels similar to the CI users.

## Materials and Methods

### A) Montreal Emotion Identification (MEI) Test

Participants were asked to judge whether musical excerpts sounded “happy” or “sad”. Stimuli consisted of four different conditions, each with 32 piano selections [17]. The first condition (“Original tunes”) contained 8-to-11 second-long musical excerpts drawn from the Western classical music repertoire (baroque, classical, romantic, and contemporary periods), so that half of the excerpts evoked a sense of happiness and the other half evoked a sense of sadness according to a priori classification: major mode and 80–255 beats-per-minute (bpm) for the happy selections, and minor mode with 20–100 bpm for the sad tunes. In the second “Mode-changed” condition, the pieces were transcribed in the opposite mode with respect to their original mode, and in the third “Tempo-changed” condition, all tempi were set to the same value of 80 bpm so that the perceived rhythm was slower for the originally happy tunes and faster for the originally sad tunes. In the fourth condition, the previous two modifications were combined (“Mode- and Tempo-changed”). Participants completed a six item training session using a different set of musical excerpts to determine their consistency in identifying emotions as happy and sad. All 128 trials were then presented with a different randomization sequence for each subject, without providing any feedback. At the end of the test session, the participants were asked if they were familiar with any of the excerpts. None recognized the musical pieces.

#### 1) Experiments 1 and 2

MEI musical excerpts were presented in a sound treated booth using Windows Media Player and a loudspeaker at 0° azimuth and 1 m from the participant at an average fixed level of 60 dB SPL (quiet condition).

#### 2) Experiment 3

Three conditions designed to degrade pitch cues were added to the quiet condition: two vocoder conditions simulating listening through a CI (MEI—vocoder) and an environmental noise condition (MEI—real world noise). Vocoded conditions were created by filtering each musical item from the original MEI test using AngelSim [88], a free online CI simulator. Two different simulations were created: a 32-channel and 22-channel CI using specific settings that matched those of our CI group in Experiment 2 (Table 1). The 32 channel vocoder represented a partial restriction in frequency/pitch and the 22 channel vocoder provided more restricted representation of frequency/pitch.

**Table 1:** Cochlear implant characteristics for CI participant inclusion and vocoder creation.

Perimodiolar Array	Speech Processor	Processing Strategy	Number of Maxima	Rate	Pulse Width	Electrodes Turned Off
CI24RE	CP810	ACE	10	900 pps	25 μs	None
CI24R(CA)						
CI513						

The real world noise condition was determined by comparing effects of white, pink, brown and speech babble noise on test accuracy of seven adults from Experiment 1. Keeping the primary signal (MEI) intensity fixed at 60 dB SPL, the level of each noise was increased until the accuracy of the unaltered melodies decreased to that of the children using a unilateral CI (defined as ~85% of accuracy; see pilot test group described below and Hopyan et al. [73]).

#### 3) Reactobutton App

An iPad application (Reactobutton) was specifically developed for the present study. This “app” synchronizes the musical stimulus onset, delivered through a computer, with the appearance of two buttons on the screen of an iPadMini. Drawings of a happy and sad face [73] were presented together with their respective emotion labels underneath. Subjects were instructed to keep the device on their laps and to push the happy face button if the tune sounded happy and the sad face button if the melody sounded sad, as soon as they knew which emotion was conveyed. Thus, reaction time was collected in addition to which emotions were selected.

Each melody in its original form had an assigned emotion provided by the MEI developers [17]. Accuracy was measured as the percent correct identification of the assigned emotion in the original melody condition. For the three conditions in which the melodies were altered, “change in opinion” was calculated as the percentage of melodies for which the children had a different emotion choice compared to the originally assigned emotion of the unaltered/original melody. Finally, reaction time represented the length of time between stimulus onset and emotion selection.

### B) Groups of children

Exclusion criteria for all three experiments included any history of neurological difficulties (e.g., previous head injuries requiring hospitalization, cerebral palsy), diagnosed psychiatric disorders, or developmental disorders known to impact emotion recognition skills. Parental written informed consent and participant assent were obtained from all participants and protocols were approved by the Hospital for Sick Children Research Ethics Board.

#### 1) Experiment 1

Study participants were 33 native English speakers with normal hearing (13 males, 20 females; age range 6.6–40.0 years), and demographic details are summarized in Table 2. All but one of the participants was right-handed, and 15 of the 33 participants (45%) had 1-to-10 years of formal music training. Each individual underwent preliminary pure tone audiometric screening to confirm bilateral normal hearing (0.25–8 kHz thresholds <20 dB HL).

**Table 2:** Demographic information for participants in Experiment 1.

Demographic Variable	
Age at test*	16.4 ± 8.3 (6.6–40.0)
Gender (Female/Male)	20/13
Adults (>18ys)	9/33
Musical Training (Yes/No)	3/9
Years of Musical Training*	9.33 ± 0.94 (8–10)
Children (<18ys)	24/33
Musical Training (Yes/No)	12/24
Years of Musical Training*	4.17 ± 2.88 (1–10)

* mean age in years ± standard deviation (range)

#### 2) Experiment 2

Children with bilateral severe-to-profound sensorineural hearing loss were enrolled who received two CIs, both simultaneously and sequentially, or only one CI but continued to wear a contralateral hearing aid (bimodal users). For inclusion children had to be: 1) between 6 and 15 years of age; 2) implanted by age three years, except for the bimodal group who were generally older when implanted due to a progressive hearing loss; and 3) using both devices for at least four years. Only children with specific device characteristics were included in order to minimize any external processor or array-related effects (see Table 1). Using these criteria, 42 child CI users were recruited from the Cochlear Implant Program at the Hospital for Sick Children: 31 bilateral CI users (9 simultaneously implanted and 22 sequentially implanted) and 11 bimodal users. All CI users received auditory-verbal therapy and orally communicated with Canadian English. About a third (38%) of this group of children received at least one year of formal musical training. As a control group, we enrolled 16 age-matched children, previously recruited for Experiment 1. Main demographic characteristics of Experiment 2 study groups are detailed in Table 3. Formal musical training was defined as a period of more than 6 months of music theory classes and practicing an instrument. Of the 12 children who had musical experience, 8 played the piano, 3 played the guitar, and 1 played both drums and the piano.

**Table 3:** Demographic information for each group of children in Experiment 2.

		Bilateral CI Users	Bimodal Users	Normal Hearing
Number of participants (Female/Male)		31 (12/19)	11 (4/7)	16 (9/7)
Number of participants with musical training (%)		14 (45%)	2 (18%)	9 (56%)
Age at test*		10.2 ± 1.8 (6.9–14.0)	10.3 ± 2.4 (6.8–14.0)	10.1 ± 2.0 (6.5–13.0)
Bilateral Deprivation*		0.6 ± 0.4 (0.02–2.0)	1.3 ± 1.7 (0.06–5.5)	N/A
Unilateral Deprivation*		2.5 ± 2.8 (0.0–9.4)	0.4 ± 0.8 (0.05–2.9)	N/A
Time in Sound (TiS)*	Electric	8.4 ± 2.0 (3.8–11.5)	3.3 ± 3.1 (0.7–11.4)	N/A
	Acoustic	0.9 ± 0.7 (0.3–2.9)	9.3 ± 2.4 (6.4–12.5)	10.1 ± 2.0 (6.5–13.0)
Musical Training*		2.9 ± 1.8 (1.0–6.0)	1.5 ± 0.5 (1.0–2.0)	2.7 ± 1.2 (1.0–5.0)

* mean in years ± standard deviation (range) Time in Sound (TiS) was defined as the duration of time the children had access to sound ≤40 dB HL averaged across 250, 500 and 1000 Hz. This included any unaided or aided hearing prior to implant as well as post-implant hearing.

#### 3) Experiment 3

Each of the 33 normal hearing individuals recruited to participate in Experiment 1 also participated in Experiment 3. Of the 33 individuals, 22 were able to complete all test conditions; 11 individuals could only reliably complete three out of four random conditions due to the highly time- and effort-consuming nature of the complete task. Data from Experiment 1 was used in Experiment 3 (quiet condition). This avoided repeating the same test in the same condition, thus reducing potential fatigue and learning effects. Demographic details of the study groups are summarized in Table 4.

**Table 4:** Demographic information for participants in Experiment 3.

	Quiet	Pink Noise	Vocoder 32	Vocoder 22
Gender (Female/Male)	33 (20/13)	29 (19/10)	31 (20/11)	29 (17/12)
Musical Training (Yes/No)	15/18	13/16	17/14	12/17
Age at test*	16.4 ± 8.3 (6.6–40.0)	16.8 ± 8.1 (6.6–40.0)	18.2 ± 8.3 (7.4–40.0)	16.9 ± 8.3 (6.6–40.0)
Years of Musical training*	5.2 ± 3.3 (1.0–10.0)	6.0 ± 3.0 (2.0–10.0)	5.8 ± 3.1 (1.0–10.0)	5.2 ± 3.1 (1.0–10.0)

* mean in years ± standard deviation (range)

#### 4) Pilot Testing for Experiment 3

We enrolled a sample of five children using a right unilateral CI but no hearing aid in the non-implanted ear. These children were aged (mean ± standard deviation) 10.1 ± 12.1 years with 7.6 ± 23.0 years of electrical hearing, and met the device characteristics and inclusion criteria for Experiment 2. The MEI was completed in the original condition, and because their accuracy with our set-up was consistent to previously reported values [73], this value was subsequently used as a baseline reference for the second part of the pilot test administered to the normal hearing sample.

### C) Analyses

SPSS version 22 [89] was used to perform statistical analyses for all experiments. Demographic features were assessed for similarity using one-way analysis of variance (ANOVA) and post-hoc t-tests with Bonferroni corrections. Simple between-group comparisons were analyzed with two-tailed independent t-tests using Bonferroni corrections for multiple comparisons. Multivariate ANOVA was used to assess accuracy and reaction time for original conditions with two between-subject factors: age (child/adult) and musical training (music/no music). To determine whether accuracy differed from chance (i.e., 50%) one-sample t-tests with Bonferroni corrections were completed. For changes in opinion and reaction time across cue change conditions, main effects and interactions were analyzed using repeated measures analysis of variance (RM-ANOVA) on complete data sets only, with cue-change condition as a within-subject factor, and age and music groups as between-subject factors. Greenhouse-Geisser corrections for sphericity were used when indicated. Bonferroni corrections were used in pairwise comparisons and post-hoc testing. To determine associations between changes in opinion and reaction time with certain factors, linear regressions were first performed by including an interaction component to determine whether associations were significantly different between two groups. If the interaction was significant at the 90% level, then a stratified analysis was performed. Otherwise, pooled associations were reported. To determine which demographic features best predicted changes in opinion with cue changes, stepwise multiple linear regression was completed with probability-of-F to enter ≤0.05 and probability-of-F to remove ≥0.1.

## Results

### A) Experiment 1

#### 1) Use of mode cues to judge emotion in music increases from childhood to adulthood and with musical experience

**Fig 1:**
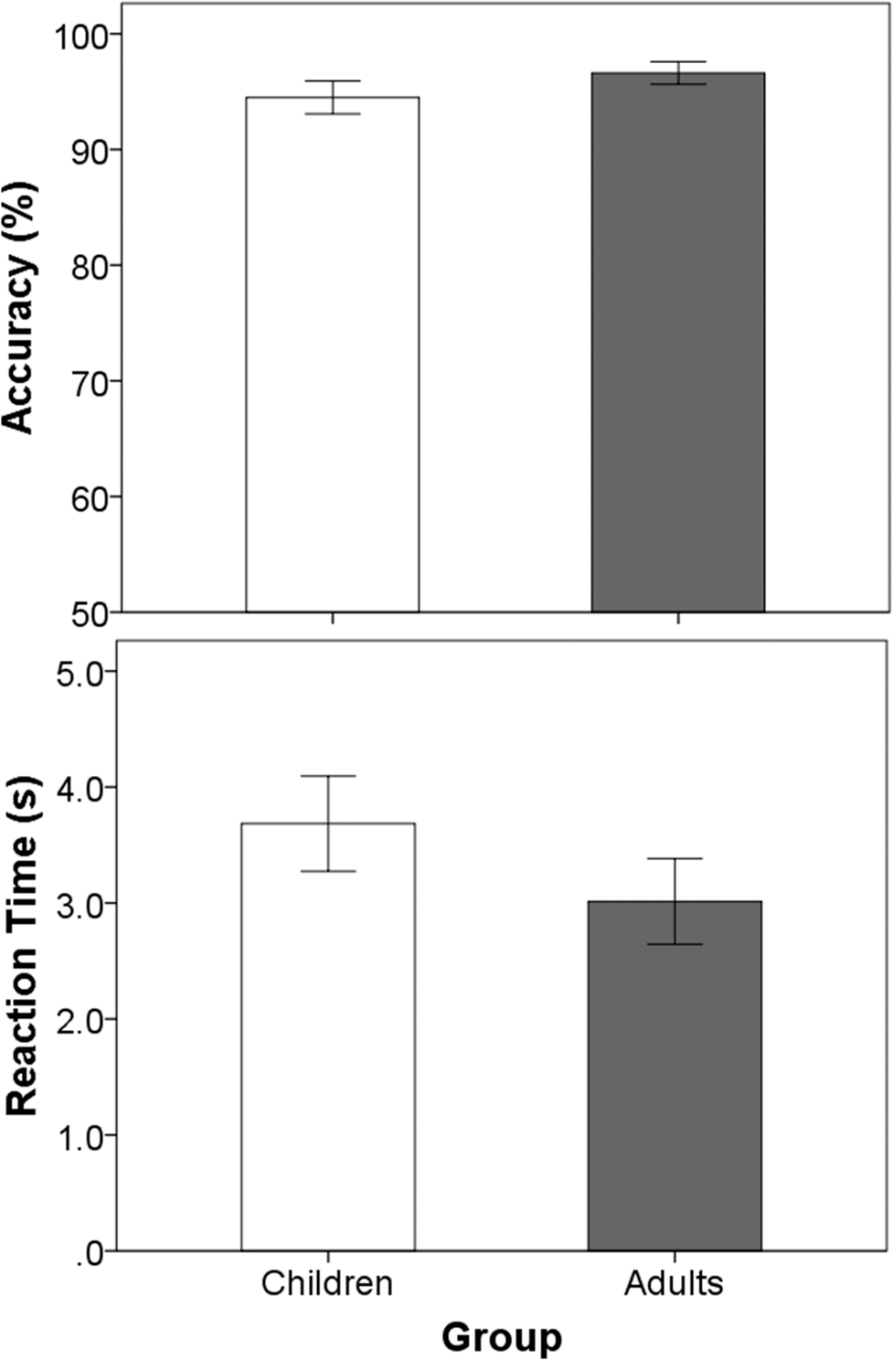
Accurate emotion identification is achieved across ages and musical experience. Mean accuracy (top) and reaction time (bottom) for the original version of the MEI test for children (white bar) and adults (grey bar) with normal hearing: both children and adults achieved similar accuracy and reaction times for distinguishing happy from sad melodies. Error bars indicate standard error.

As shown in Fig 1, both children and adults achieved similar accuracy distinguishing happy from sad melodies (t(31) = -1.04, p = 0.31). As shown in Fig 1, both children and adults achieved similar accuracy distinguishing happy from sad melodies (F(1,29) = 0.66, p = 0.42). There was no main effect of music training (F(1,29) = 1.6, p = 0.22) or interaction between age and music (F(1,29) = 0.11, p = 0.74). Furthermore, reaction times for identifying emotions in the melodies were similar for children and adults (F(1,29) = 0.35, p = 0.56), regardless of whether they had music training (age*music interaction: F(1,29) = 0.25,p = 0.62).

**Fig 2:**
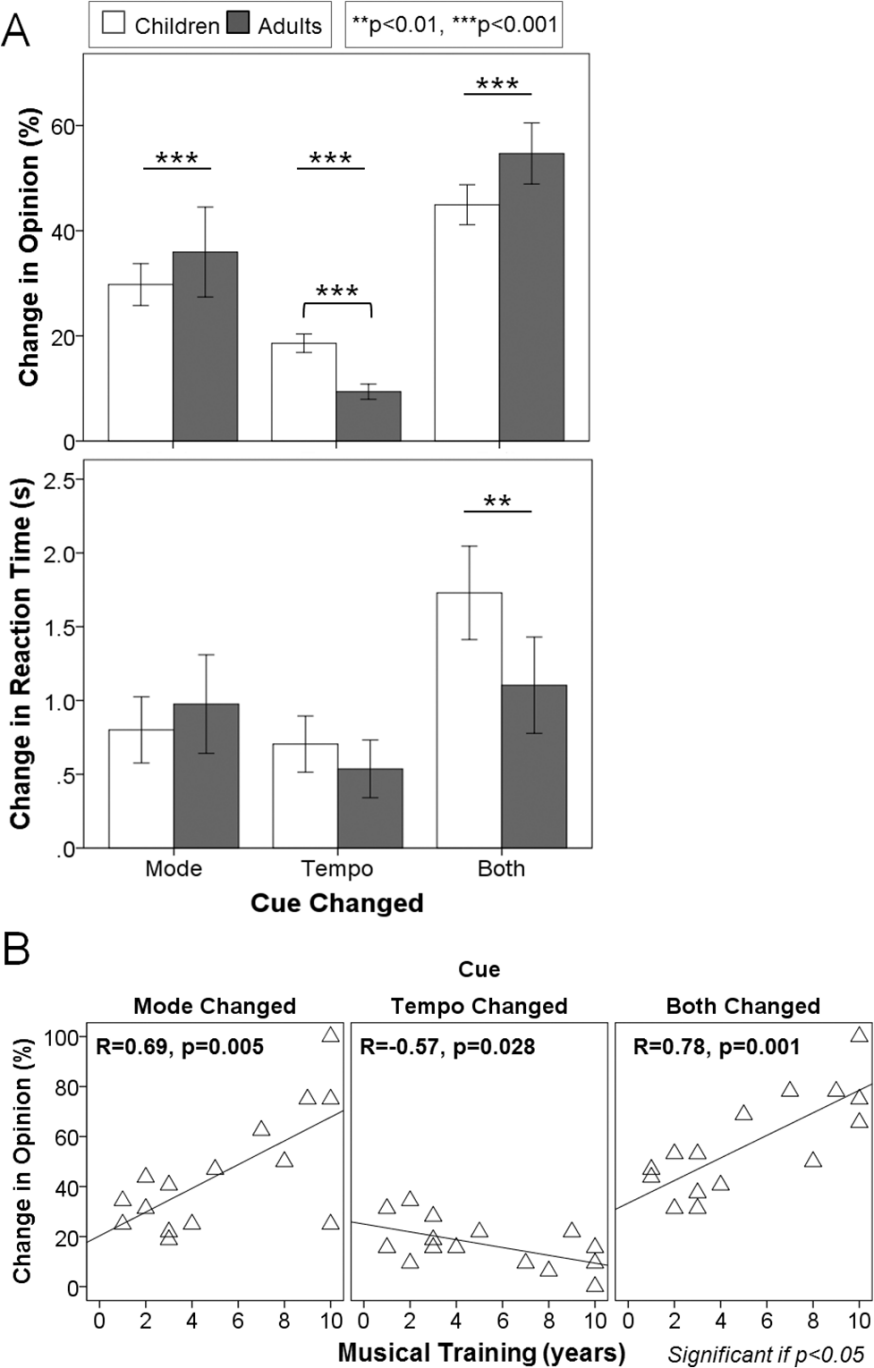
Age and musical training affect emotion identification skills. (A) Percentage change in opinion (top) and reaction time (bottom) for children (white bar) and adults with normal hearing (grey bar) when mode and tempo cues were changed: tempo changes affected opinion the least while combined cue changes affected opinion the most, though children were more affected by tempo changes than adults. (B) Effects of years of musical training in both children and adults: musicians changed their opinions 4.7% more, 1.6% less and 4.5% more with each extra year of musical training when mode, tempo or both cues were changed, respectively. Error bars indicate standard error. ** p<0.01, *** p<0.001, only associations and interactions that reach a trend level (p<0.1) or significance (p<0.05) are shown.

On the other hand, when presented with manipulated melodies individuals with normal hearing tended to change their opinion of which emotion was conveyed by the tunes relative to the original versions. When either mode or tempo changed, accuracy continued to remain above chance (Mode change: 67.6 ± 4.2%, t(32) = 4.2, p<0.0001; Tempo change: 87.6 ± 21.2%, t(32) = 31.0, p<0.0001). In contrast, once both cues were changed concurrently, accuracy reduced to chance (50.8 ± 3.6%, t(32) = 0.21, p = 0.83). The amount of change, measured by the difference in percent responses judged based on the originally intended emotion, depended on the altered cue (F(1.4, 41.9) = 67.5, p<0.0001, η_p_^2^ = 0.70). Mean data shown in Fig 2A indicated that although accuracy remained above chance, opinion significantly changed from the original condition when mode and tempo were changed independently (mode p<0.0001; tempo p<0.0001) and when both cues were changed together (p<0.0001). Overall, tempo changes affected opinion the least while combined cue changes affected opinion the most. Positive changes in RT were found in all 3 experiment conditions, reflecting the longer reaction times to respond relative to the original condition. Both adults and children took longer to make a decision when both mode and tempo cues were changed together versus changes to either cue alone (mode p = 0.04; tempo p<0.0001), but their changes in reaction times were similar with mode and tempo changes (p = 0.35).

Although there was no overall main effect of age group (F(1,29) = 2.2, p = 0.15, η_p_^2^ = 0.07), children and adults reacted differently to the different manipulations (F(1.4, 41.9) = 8.4, p = 0.002, η_p_^2^ = 0.22). Tempo changes invoked the greatest difference in behavior across development; children were more affected by these tempo changes than adults (p = 0.004). This greater use, or weighting of mode cues into adulthood was accentuated in those individuals with musical training (age*music interaction p = 0.02). Opinion changes were significantly associated with age in musicians upon mode changes (R = 0.67, p = 0.006) and combined mode and tempo changes (R = 0.60, p = 0.02), but not in individuals without musical training (mode alone: R = 0.11, p = 0.67; mode and tempo: R = 0.23, p = 0.35). Because whether an individual had musical training affected decisions regarding the emotion of a musical excerpt when cues were changed, we explored whether the amount of training was an important factor. As shown in Fig 2B, opinion changes correlated with musical training when mode and tempo cues were changed independently (mode R = 0.69, p = 0.005; tempo R = -0.57, p = 0.03) and together (R = 0.78, p = 0.001). Specifically, musicians’ opinions based on mode increased at a rate of 4.7% while the influence of tempo cues decreased by 1.6% per year. When both cues were changed, opinion change increased by 4.5% with each extra year of musical training. Consequently, changing mode cues reversed responses of the most experienced musicians but did not cause more than a 50% change in response for musicians with less than about 4 years of musical training.

### B) Experiment 2

#### 1) Children with cochlear implants abnormally weigh tempo over mode cues for judging emotion in music

**Fig 3:**
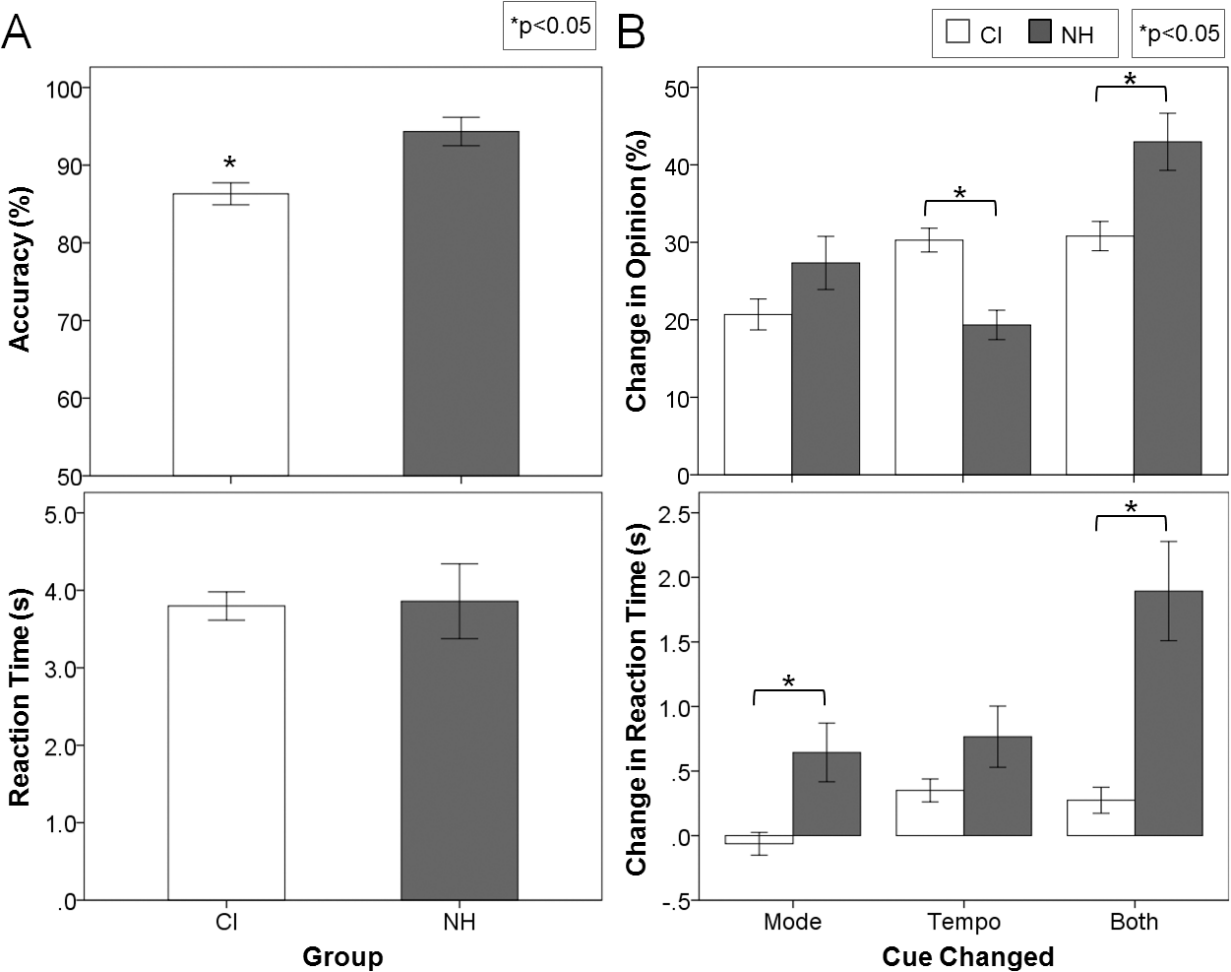
CI children rely mostly on tempo cues to detect emotions in music. (A) Mean accuracy (top) and reaction time (bottom) for child CI users (white bar) and normal hearing children (grey bar) for the original version of the MEI test: although well above chance and with similar reaction times, CI children identified the emotions with significantly less accuracy than their normal hearing peers; (B) Percentage change in opinion (top) and change in reaction time (bottom) for CI users (white bar) and normal hearing children (grey bar) for the other three experimental conditions: implanted children changed their opinion to a greater extent when tempo changed, while children with normal hearing were affected more by a concurrent change in mode and tempo. Error bars indicate standard error. * p<0.05

Children using CIs identified the emotion of the original musical excerpts with significantly less accuracy (-8.0 ± 2.6%) than their normal hearing peers (Fig 3A top plot; F(1,56) = 9.9, p = 0.003), although they still performed well above chance (mean ± SE, 86.3 ± 1.4%, t(41) = 25.7, p<0.0001). On the other hand, both groups required the same amount of time to make their decisions (Fig 3A bottom plot; 3.9 ± 0.4 s for normal hearing children and 3.8 ± 40.2 s for CI users; F(1,56) = 0.02, p = 0.88).

As observed in experiment 1, children with normal hearing retained accuracy above chance with altered mode (73.2 ± 3.6%, t(15) = 6.4, p<0.0001) and tempo (84.0 ± 1.5%, t(15) = 23.0, p<0.0001) cues but responded at chance (56.8 ± 3.9%, t(15) = 1.8, p = 0.10) with both cues changed together. On the other hand, implanted children retained accuracy above chance regardless of whether each cue was changed separately (Mode change: 81.5 ± 1.9%, t(41) = 17.0, p<0.0001; Tempo change: 71.3 ± 1.9%, t(41) = 11.0, p<0.0001) or together (Both changed: 65.3 ± 1.8%, t(41) = 8.7, p<0.0001). Changes in opinion as mode and tempo cues were altered are shown in the top plot of Fig 3B. The significant change in opinion (F(2,108) = 35.0, p<0.0001, η_p_^2^ = 0.39) occurred differently between the implanted and normal hearing groups (F(2,108) = 24.4, p<0.0001, η_p_^2^ = 0.31). There were no overall effects of group (F(1,54) = 1.4, p = 0.25, η_p_^2^ = 0.02) or musical experience (F(1,54) = 0.8, p = 0.76, η_p_^2^ = 0.002) on opinion changes; however, there was an interaction between group and music (F(1,54) = 7.2, p = 0.01, η_p_^2^ = 0.12). To explore the interaction between cue change and group further, post hoc analyses were conducted using independent t-tests with Bonferroni corrections. All children reacted similarly when mode changed (t(56) = -3.5, p = 0.27), but implanted children changed their opinion to a greater extent when tempo changed (t(56) = 4.0, p = 0.001). On the other hand, children with normal hearing were affected more by a concurrent change in mode and tempo (t(56) = -3.2, p = 0.007).

Reaction times (Fig 3B, bottom plot) increased to a significantly greater extent in children with normal hearing than children with CI when cues were changed in the melodies (F(1,54) = 23.0, p<0.0001, η_p_^2^ = 0.30), and there was a significant main effect of which cue changed (F(2,108) = 29.4, p<0.0001, η_p_^2^ = 0.35). More specifically, reaction times changed significantly as each cue was changed, either independently (p = 0.049) or in conjunction (p<0.0001). When collapsed across group, changes to both cues affected reaction time the most, then tempo changes, and finally mode changes. Furthermore, there was a significant two-way interaction between cue and group (F(2,108) = 17.6, p<0.0001, η_p_^2^ = 0.25). Children with normal hearing took longer to make decisions when mode was changed (t(56) = -3.5, p = 0.002) and when both cues were changed together (t(56) = -4.1, pc = 0.002), but not when tempo was changed alone (t(56) = -2.0, p = 0.14), although there was a similar trend for this cue change as well.

#### 2) Judgment of emotion in music is fairly stable during childhood regardless of musical training

**Fig 4:**
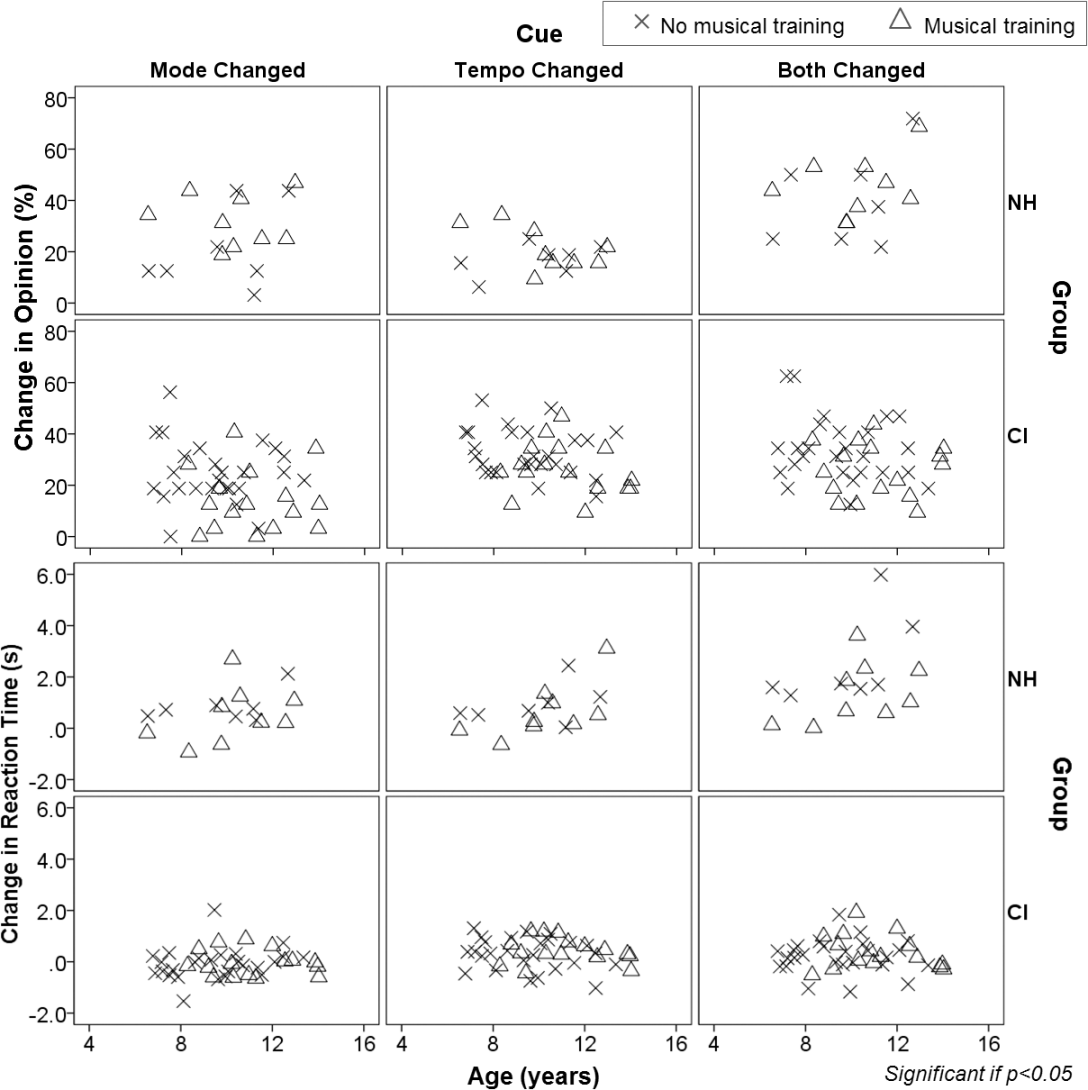
Musical training has little impact on emotion identification skills in children using CIs. Percentage change in opinion and reaction times when cues are changed across age and in those with (triangles) and without (crosses) musical training in children using CIs and with normal hearing (NH). Musical experience did not significantly affect responses or reaction times across all cue changes in CI children. Children with normal hearing who had musical training grew less affected by tempo changes (i.e., relied more on mode than tempo cues) as they aged. Only associations and interactions that reach significance (p<0.05) are shown.

Fig 4 shows the effects of age and musical experience on changes in opinion and reaction times of children using cochlear implants and their hearing peers. Within this group, the results are somewhat different than in the wider age range included in Fig 2B. In the group of CI children shown in Fig 4, musical experience did not impact responses or reaction times across all cue changes (music*cue interaction [change in opinion/change in reaction time]: mode p = 0.83/0.29, tempo p = 0.93/0.79, both p = 0.53/0.53). The same held true for normal hearing peers when mode changed either independently or with concurrent changes in tempo; however, when tempo cues changed, normal hearing children with musical training were less affected by tempo changes (ie. relied more on mode than tempo cues) as they aged (music*cue interaction [change in opinion/change in reaction time]: mode p = 0.35/0.79, tempo p = 0.04/0.33, both p = 0.73/0.54). We assessed this interaction more closely by stratifying both groups according to whether or not they had musical training (Fig 4). When tempo cues were altered, opinion changes were not significantly associated with age in non-musicians (R = -0.49, p = 0.26) and only reached a trend level of significance in musicians (R = -0.60, p = 0.09).

#### 3) Cochlear implant users rely more on tempo cues when implanted earlier in life and less when they have access to acoustic hearing

Given that age and musical training did not explain why children using cochlear implants abnormally relied on tempo cues, additional factors related to time were explored. Auditory deprivation was not significantly associated with opinion changes across cue changes, regardless of whether the deprivation was unilateral (mode R = -0.14, p = 0.37; tempo R = 0.25, p = 0.10; both R = -0.05, p = 0.75) or bilateral (mode R = -0.25, p = 0.11; tempo R = -0.24, p = 0.12; both R = -0.06, p = 0.69), and regardless of whether the children were bimodal or bilateral CI users (group*deprivation interaction [bimodal/bilateral CI]: mode p = 0.63/0.16, tempo p = 0.41/0.99, both p = 0.61/0.18).

**Fig 5:**
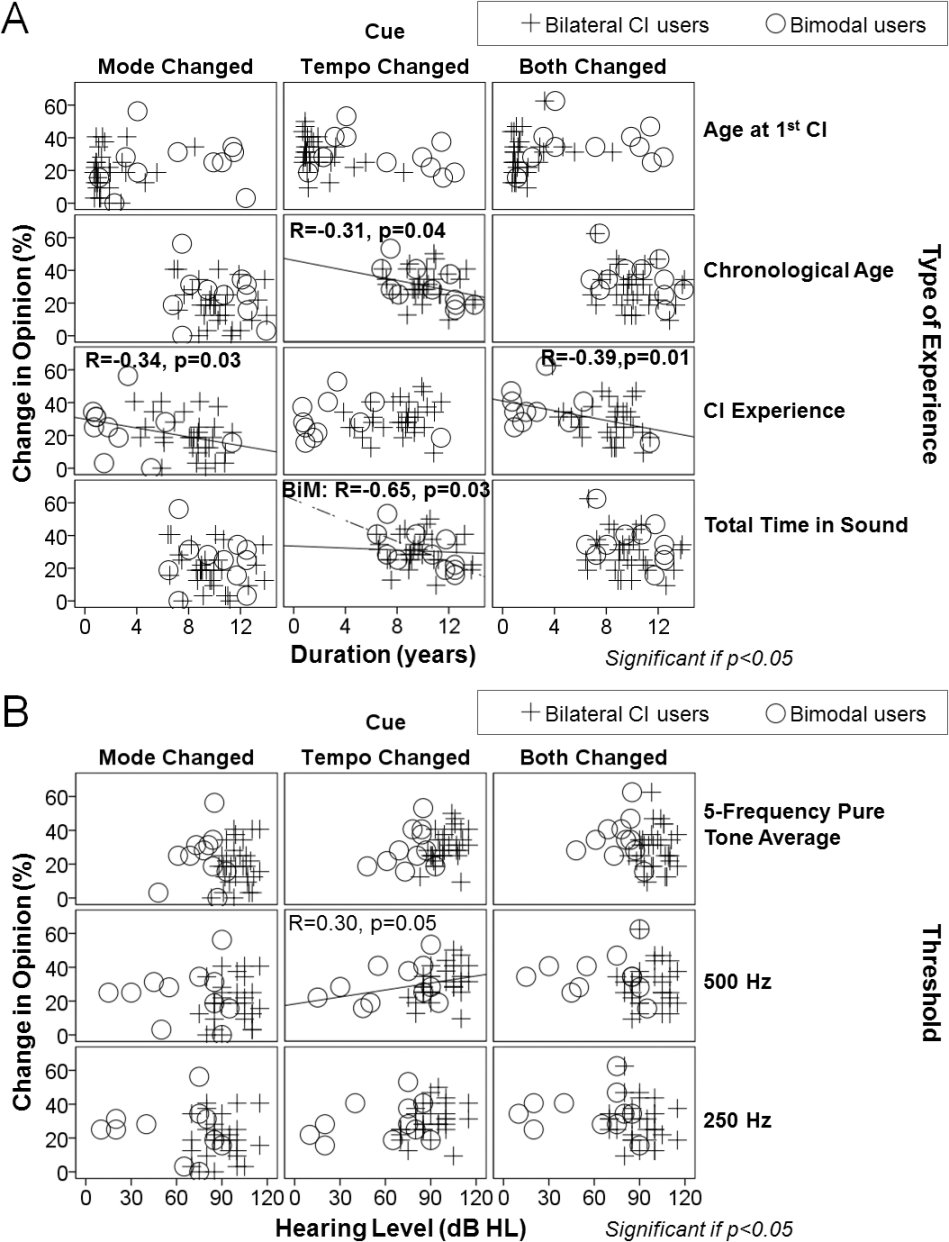
Role of duration and type of hearing experience in CI patients' emotion identification skills. (A) Percentage change in opinion when cues are changed across duration and type of hearing experience in bilateral CI users (plus signs) versus bimodal users (circles): hearing experience rather than absolute age seems to be the most important factor for developing emotion identification in music. Some of the main associations are as follows: length of hearing deprivation did not affect emotion identification skills; the longer children used CI(s), the less they were affected by changes in mode cues and the more they relied on tempo; children with greater acoustic sound experience (bimodal users) were less affected by tempo cues than the bilateral CI users; age at first implantation and age at test did not affect overall performance. (B) Percentage change in opinion when cues were changed across hearing level and pure tone thresholds in bilateral CI users (plus signed) versus bimodal users (circles): children with a greater degree of low frequency hearing loss and overall hearing loss were more affected by tempo cues (ie. relied more on tempo than on mode cues). Only associations that reach a trend level (p<0.1) or significance (p<0.05) are shown.

Factors related to duration of cochlear implant input were then explored for their relationship with changes in opinion (Fig 5A). Total years of CI experience was significantly negatively associated with opinion changes, both when mode was changed independently (R = -0.34, p = 0.03) or in conjunction with tempo changes (R = -0.39, p = 0.01), indicating that children with more CI experience were using mode cues less to inform their decisions. Tempo cues were used less (as measured by opinion change) with chronological age (tempo: R = -0.31, p = 0.04). However, these associations may be confounded by other factors, such as length of pre-implant acoustic experience and age of implantation. In fact, there was a significant interaction between bilateral CI and bimodal users for total time in sound (“hearing age”) when tempo cues were changed (group*time-in-sound interaction: tempo p = 0.06), but not when tempo changed with mode or when mode cues were changed (group*time-in-sound interaction: mode p = 0.80, both p = 0.79). When stratified, bilateral CI users changed their opinions similarly for changes in tempo cues regardless of their total time in sound (R = 0.06, p = 0.74), which was predominantly electrical hearing for these children. Accordingly, total electrical hearing was not significantly associated with changes in opinion with tempo changes (R = 0.11, p = 0.48). In contrast, bimodal users with greater total hearing experience were less affected by tempo changes (ie. relied more on mode cues) (R = -0.65, p = 0.03). These children had a longer period of bilateral acoustic experience, as reflected in their later age at implantation (Fig 5A, middle top panel). Age at implantation was also associated with opinion changes with tempo changes, albeit to a lesser extent (R = -0.29, p = 0.07).

Due to the difference in response to tempo cues between bilateral and bimodal users with the type of hearing experience, we focused on the potential role of residual hearing in changing opinions (Fig 5B). Opinions changed similarly between bilateral CI and bimodal users, despite differing pre-implant acoustic hearing thresholds (group*threshold interaction [250 Hz/500 Hz/5-frequency average]: mode p = 0.68/0.71/0.89, tempo p = 0.53/0.34/0.76, both p = 0.51/0.96/0.98). However, when considered together, children with a greater degree of low frequency hearing loss (i.e., poorer hearing thresholds) were more affected by tempo cues (ie. relied less on mode cues) (R = 0.30, p = 0.05).

### C) Experiment 3

#### 1) Normal reliance on mode cues to judge emotion in music persists in noise but not after simulated cochlear implant processing

Both vocoded acoustic input and noise were used to acutely degrade acoustic musical input in order to study which musical components normal hearing individuals would use to identify emotion. Four different noise conditions were first piloted with group of normal hearing adults (n = 7). Noise was presented at the highest level which did not decrease accuracy of performance of emotion identification of music in its original condition. Of all four noise conditions, only pink noise significantly: 1) decreased accuracy when tempo cues were changed in individuals with normal hearing (p = 0.02), and 2) decreased accuracy in the tempo changed condition to the point that scores were not significantly different from that of 5 children with unilateral CI use listening to the original condition in quiet (p = 0.24). In all other noise conditions, accuracy was better than the best listening condition in children with unilateral cochlear implants (white noise p = 0.014; brown noise p = 0.001; babble noise p = 0.002). Therefore, pink noise was used along with the two vocoders to test which cues a larger group of normal hearing individuals use to identify emotion in music.

**Fig 6:**
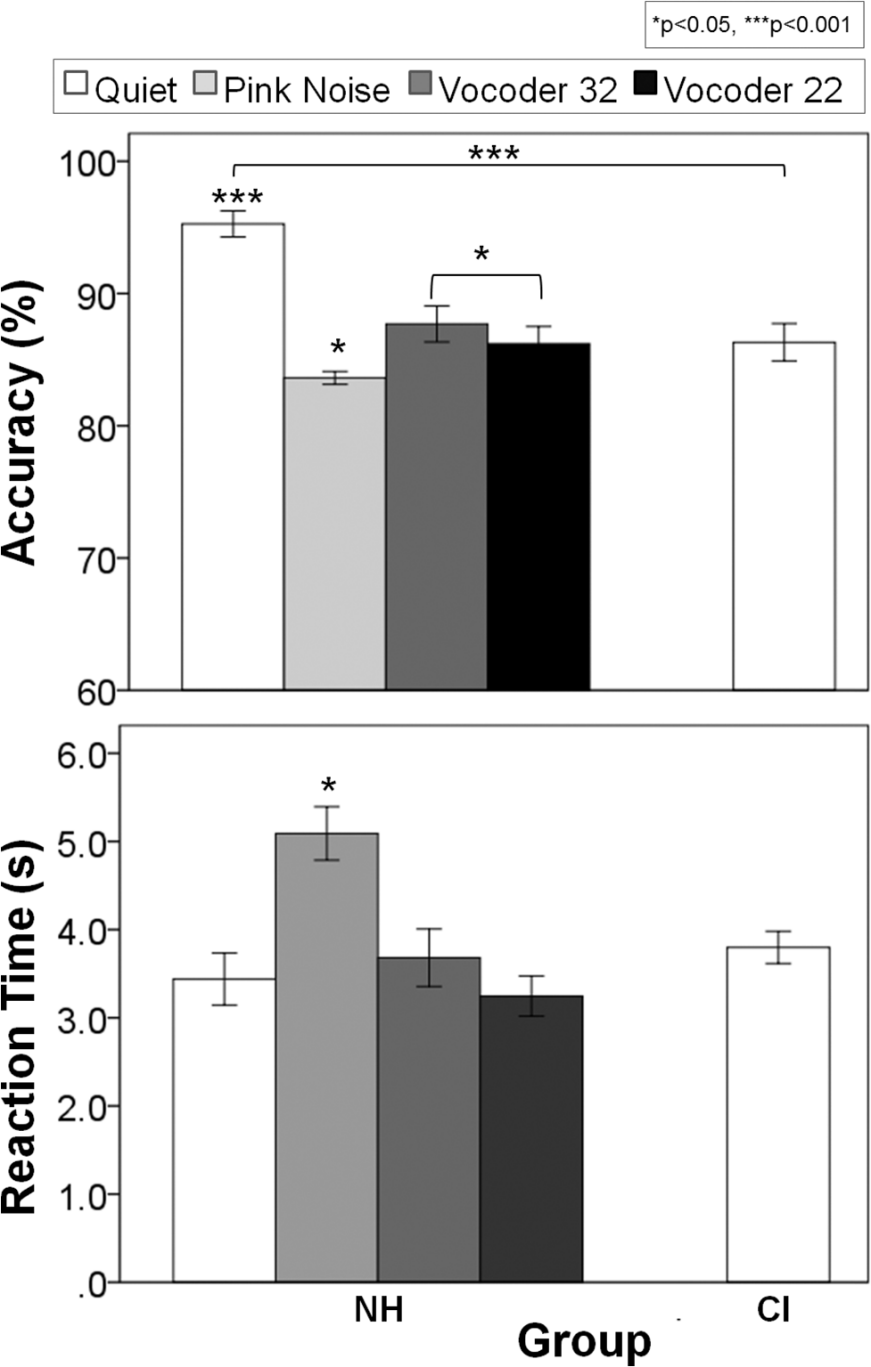
Emotion identification under different pitch-degraded conditions in normal hearing subjects. Mean accuracy (top) and reaction time (bottom) for the original version of normal hearing subjects under different acutely degraded acoustic input conditions, compared to those of a group of unilaterally implanted CI children in quiet (right white bar): accuracy in the pink noise (light gray), vocoder 32 (dark gray) and vocoder 22 (black) conditions were significantly less than in quiet (white), with pink noise generating the worst performance and the longest reaction time. Error bars indicate standard error. * p<0.05, *** p<0.001.

Effects of acutely degrading acoustic input through pink noise and partial (32 channel) and more restrictive (22 channel) vocoders are shown for normal hearing groups and the comparative CI group in Fig 6. Accuracy (F(2.0,41.8) = 31.2, p<0.0001, η_p_^2^ = 0.60) and reaction time (F(2.1,44.1) = 18.6, p<0.0001, η_p_^2^ = 0.47) for the original melodies were significantly different across conditions. Pairwise comparisons using Bonferroni corrections indicated that accuracy in the presence of pink noise and in the two vocoder conditions was significantly less than in quiet (p<0.0001). Vocoder conditions resulted in similar accuracy (p = 1.0), but surprisingly, the pink noise condition generated the worst performance compared to both vocoder conditions (vocoder 32: p = 0.04; vocoder 22: p = 0.02). Furthermore, reaction time was significantly longer in the presence of pink noise than any other condition (p<0.0001), and this was the only condition in which reaction times of normal hearing individuals were different than child CI users (t(69) = 3.9, p = 0.001). As in Experiment 2, individuals with normal hearing performed better than the CI users (p<0.0001); however, this difference/advantage was lost in all three degraded conditions (pink noise p = 0.31, vocoder 32 and 22 p = 1.0). An average signal to noise ratio of -12 ± 2 dB was required to degrade normal hearing individuals’ performance to that of CI users.

**Fig 7:**
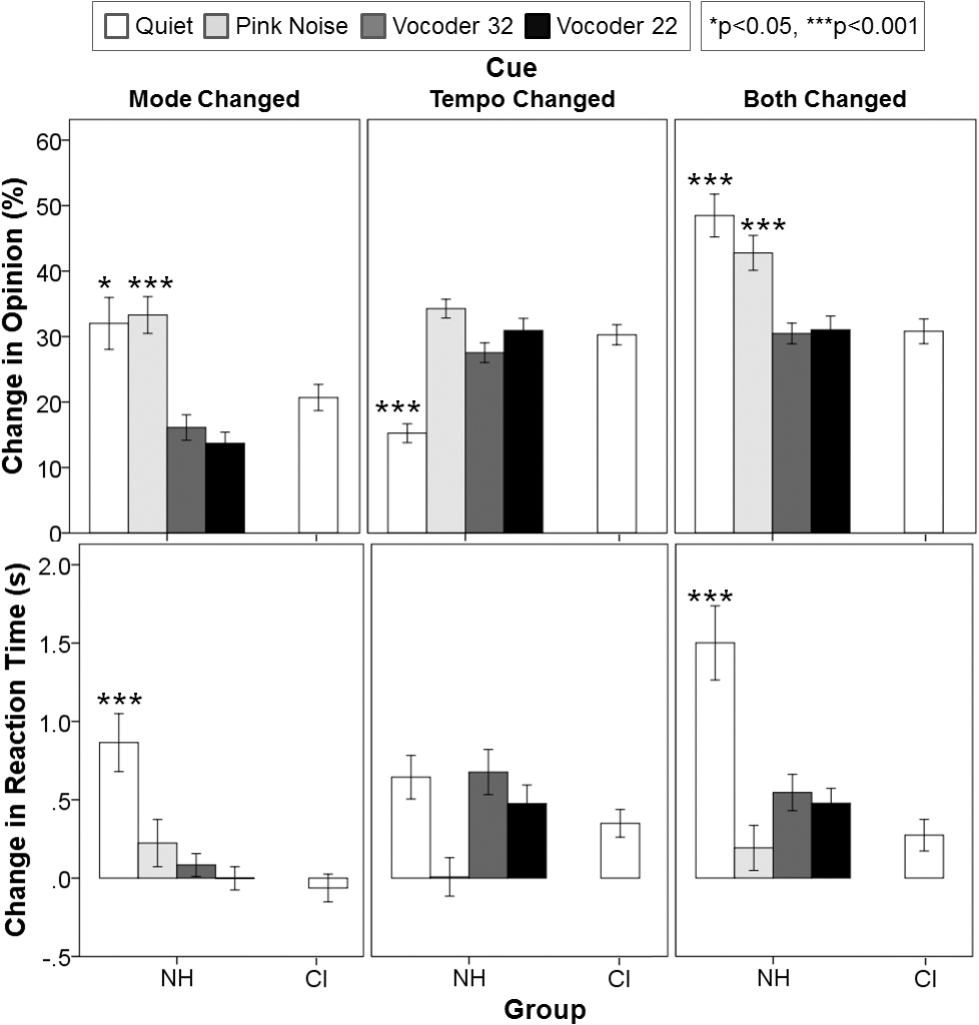
Normal hearing subjects attempt to use mode cues whenever possible. Percentage change in opinion (top) and change in reaction time (bottom) in response to cue changes for normal hearing subjects (NH) listening to degraded conditions compared to unilateral CI children (CI) listening in quiet: children with normal hearing performed similarly to unilateral CI users in both the vocoder 32 (dark gray) and vocoder 22 (black) conditions across cue change conditions, but also in the pink noise condition (light gray) only when tempo cues were changed. They still performed similarly in the pink noise and quiet (white) conditions when mode cues were changed independently or in conjunction with tempo cues. Error bars indicate standard error. * p<0.05, *** p<0.001.

Changes in opinion with cues changes for each of the signal degraded conditions are shown in Fig 7. There were simple main effects of condition (F(2.3,48.9) = 16.4, p<0.0001, η_p_^2^ = 0.44) and cue changed (F(1.3,26.8) = 24.8, p<0.0001, η_p_^2^ = .54) on these opinion changes in individuals with normal hearing. Pairwise comparisons indicated that overall, opinion changes were similar in quiet and pink noise conditions (p = 0.49) but these two conditions were significantly different than both vocoder 32 (quiet p = 0.01, pink noise p<0.0001) and vocoder 22 conditions (quiet p = 0.02, pink noise p<0.0001), which were similar to one another (p = 1.0). Furthermore, opinion changes were significantly greater when both cues were changed (p<0.0001) than either cue changed alone (p = 1.0). However, individuals with normal hearing still responded differently across cue changes in the different degraded conditions (F(3.0,63.5) = 27.1, p<0.0001, η_p_^2^ = 0.56). When mode cues were changed independently or in combination with changes in tempo cues, normal hearing individuals changed their opinions significantly more than CI children in quiet (mode p = 0.10, both p<0.0001) and pink noise (mode p = 0.007, both p = 0.007), but they behaved similarly to CI children in the vocoder 32 (mode p = 1.0, both p = 1.0) and vocoder 22 (mode p = 0.12, both p = 1.0) conditions. Conversely, when tempo cues were changed, individuals with normal hearing changed their opinions significantly less than children with CIs only in quiet (tempo p<0.0001), but not in pink noise (p = 0.74) or vocoder 32 (p = 1.0) and 22 (p = 1.0) conditions. With respect to changes in reaction times with these decisions, there were also significant main effects of condition (F(1.9,40.7) = 7.1, p = 0.003, η_p_^2^ = 0.25), cue (F(1.9,40.5) = 15.2, p<0.0001, η_p_^2^ = 0.42), and a two-way interaction between condition and cue (F(4.3,90.3) = 7.2, p<0.0001, η_p_^2^ = 0.25). Pairwise comparisons indicated that reaction times of individuals with normal hearing were significantly longer in quiet than any other condition (pink noise p = 0.03, vocoder 32 p = 0.04, vocoder 22 p = 0.01), regardless of which cues were changed. It was only in the quiet condition that individuals with normal hearing took significantly longer than CI children to make decisions, specifically when mode cues were changed independently (p<0.0001) or in conjunction with tempo changes (p<0.0001).

#### 2) Mode cues to judge emotion in music were used increasingly by individuals with long term musical training in degraded conditions

**Fig 8:**
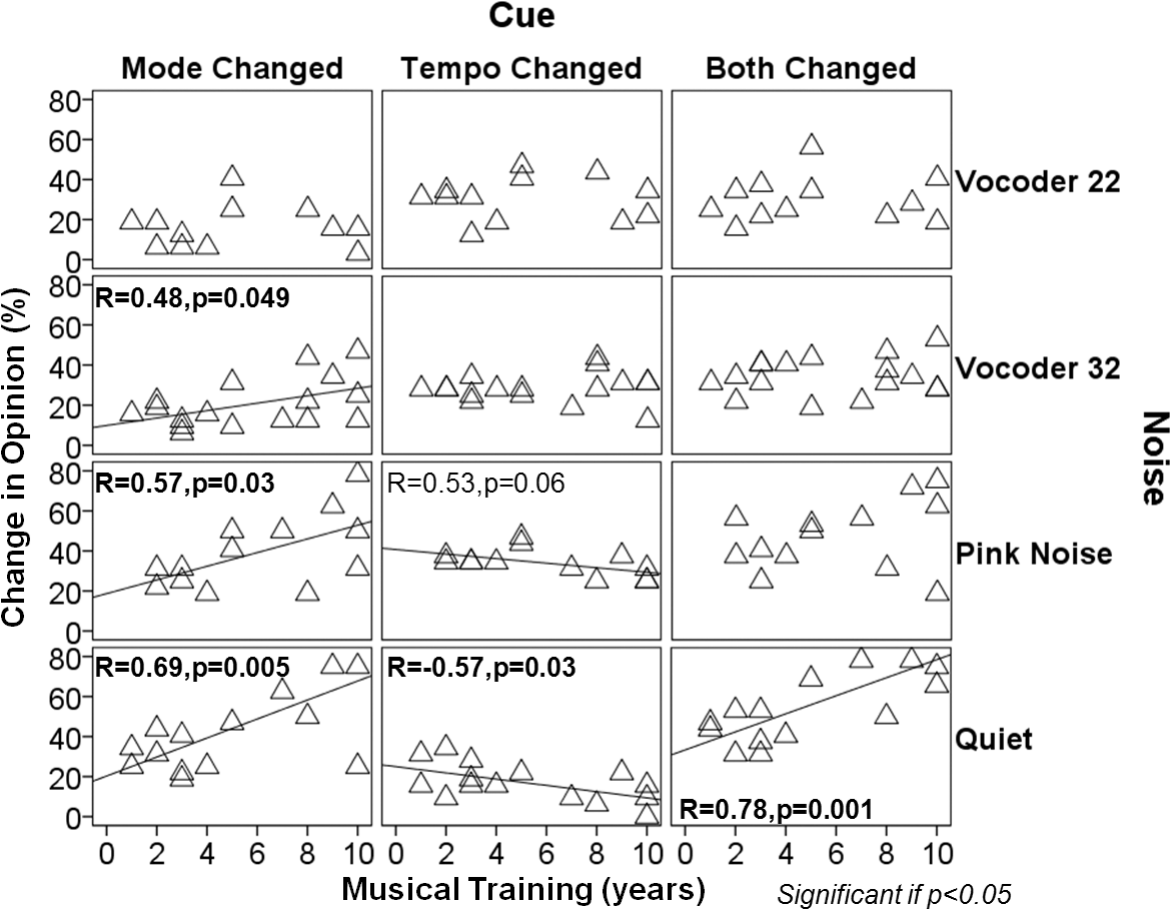
Musical training enhances use of distorted mode cues. Percentage change in opinion when cues are changed across years of formal musical training and experimental conditions in musicians: music experience did not affect reliance on tempo, even with a pitch-deprived signal, while specific training progressively affected emotion identification skills when pitch cues were present. When little-to-no pitch information was provided, length of musical training had no bearing on opinion changes with mode changes. Only associations that reach a trend level (p<0.1) or significance (p<0.05) are shown.

Effects of musical training were explored for each degraded condition as well. Individuals with musical training reacted differently as they aged (chronological age) when mode cues changed than those without musical training (cue*music group interaction p = 0.02): opinions changed significantly more with age in musicians (R = 0.67, p = 0.01) but non-musicians responded similarly regardless of their age (R = 0.10, p = 0.67). Once pitch cues were masked or degraded, these associations became non-significant, with the exception of the interaction between musical experience and age when mode cues were changed in the vocoder 32 condition (music*age interaction p = 0.01; No musical training group R = 0.34, p = 0.22; Musical training group R = 0.55, p = 0.02). Additionally, there was a trend that reaction times lengthened with age when individuals with normal hearing listened to the vocoder 32 musical excerpts with altered mode cues (R = 0.34, p = 0.06). In most conditions, as musical training increased (Fig 8) opinions changed significantly more with mode changes, although the degree to which opinions changed became less significant as pitch cues were progressively masked or removed: in quiet (p = 0.005), pink noise (p = 0.03), vocoder 32 (p = 0.05), and vocoder 22 (p = 0.97) conditions. On the other hand, there was only a significant association between opinion changes and musical training in quiet when both mode and tempo were changed in conjunction (R = 0.78, p = 0.001). Although the pattern was similar across conditions, opinions conversely changed less with musical training in quiet (R = -0.57, p = 0.03) and pink noise (R = -0.53, p = 0.06) when tempo cues were changed.

## Discussion

### A) Experiment 1: Effects of musical training for judging emotion in music

#### 1) Accurate judgment of emotion in music is achieved across ages and musical experience

The results from the present experiments confirmed previously reported findings that children as young as six years and adults as old as 40 years had similarly excellent accuracy identifying basic emotions in music [2]. Neither age nor formal musical training affected scores which were near ceiling levels (95.3 ± 5.6%). Participants were asked to respond as quickly as possible, and were able to make highly accurate decisions after hearing only 3.4 ± 61.7 s of the music on average (~ a third of the piece). Nonetheless, the time required to make these decisions tended to decrease with age (Fig 1). This relationship was consistent with previous reports of decreasing reaction times in older children with normal hearing on tasks of binaural fusion [78], and may be related to changes in cortical connections that occurs in normal auditory development [90, 91] as well as a progressive experience with music over time.

#### 2) Age and musical experience accentuates normal reliance on pitch cues

An advantage of the MEI test is that it allowed for separate analyses of the relative importance of two major contributors to emotion identification in music, namely mode and tempo. The assumption was that if opinion from the original version did not change when one of the two cues was altered, it was likely that the second, fixed cue contributed more to labelling the emotion conveyed by the melody. In Fig 2A, the changes in opinion are plotted when mode, tempo or both cues were altered in the original excerpt. Although both adults and children showed changes in opinion to these alternate conditions, mode changes caused greater opinion changes than tempo changes. This behavior was exaggerated in adults, who relied on pitch cues more heavily than the children did. These findings are consistent with the previous literature [2]. This might reflect an earlier stage of development for the use of both cues in children. When both cues were changed, the original emotional content of the melodies was relatively neutralized, and the choices for happy or sad emotion reduced to chance levels for both children and adults, accordingly. Interestingly, there was no complete reversal of opinion (i.e., scores near 0%). This finding is consistent with previous studies in normal hearing listeners [17] and CI users [73], indicating that there are additional components to the music other than mode and tempo which aid emotion identification [17]. Overall reaction times were similar for adults and children. They both took an average of 3 seconds from the onset of the musical stimulus to select 1 of the 2 emotions. The time needed to choose whether the tunes sounded happy or sad did not change significantly when either mode or tempo were altered separately, although more time was needed to identify the emotion when both cues were changed at the same time. This effective neutralization of the original music caused children to take longer additional time to decide than adults, reflecting a higher reliance on both mode and tempo cues to judge emotion in music and/or an increased difficulty of the task for children.

Musical training is known to enhance many neural networks involved in music processing. Kraus and colleagues found that training-related plasticity leads to neuroanatomical differences and improves neural encoding of sound [92]. Both structural and functional changes in the brains of musicians have been found [30–34, 36, 93, 94]. Musical exposure also enhances pitch discrimination and melody contour identification [95]. The present study adds to these findings by showing a significant interaction between age and musical experience. As shown in Fig 2B, opinion changes induced by mode alteration were exaggerated by musical training. This implies that musicians were affected to a greater extent by mode changes than non-musicians as they aged. Taken together, musical experience primarily enhanced the ability to analyze the interval between notes: the older musicians became, the more they identified happy tunes with major chords and sad melodies with minor chords, regardless of their tempi. The reaction time data suggested that individuals with normal hearing took about the same amount of extra time to make a decision when mode, tempo or both cues were changed, regardless of age or musical experience. This implied that the emotion identification was equally fast, even though the relative importance of each strategy may have differed in terms of musical experience between groups. The greater reliance on tonality was affected by the overall duration of continuous musical training, regardless of chronological age. In fact, with every year of musical training opinions were increasingly affected by mode and less affected by tempo. The specific age at which music training began was not a significant factor in this cohort. Interestingly, the difference between musicians and non-musicians became larger starting from late adolescence, despite early commencement of musical training. This could mean that musical exposure played a major role during childhood in terms of development of neural networks with effects appreciated only later in life [25, 56].

### B) Experiment 2: Cochlear implant listening impairs perception of emotion in music

#### 1) Cochlear implant users rely on tempo to judge emotion in music

The unrefined spectral representation provided by currently available cochlear implants is adequate to represent speech signals, as evidenced by acquisition of oral communication in early implanted children who undergo appropriate auditory verbal rehabilitation [96, 97]. However, poor spectral representation provided by CIs proved highly unsatisfactory for music which, being less redundant than language, requires a much more precise and detailed processing of the spectral aspects of the stimulus. It is clear that children using unilateral CIs hear rhythm better than tonal aspects of music [73] and use tempo cues to judge the emotion in music [75], but these skills could be different in children using bilateral CIs or in children who have considerable residual hearing and use a CI combined with acoustic hearing in the other ear (bimodal users). As shown in Fig 3A, children using CIs identified emotions conveyed by original melodies with high accuracy (mean ± SE, 86.3 ± 1.4%) but did not score as high as their peers with normal hearing (94.3 ± 1.8%). Data in both groups were similar to the cohorts in our previous study [75]. Children with CIs reacted differently from their normal hearing peers when cues were changed (Fig 3B). In contrast to the normal reliance on mode cues, CI users changed their opinions most when tempo changed, indicating the importance of tempo cues for choosing emotion in music. Adding mode changes to the tempo changes had little effect on opinion changes, again highlighting a greater weighting to tempo cues by CI users. The increased reliance on tempo cues in children using bilateral CIs was consistent with findings in children using unilateral CIs [75]. The changes in unilateral CI users were similar for both happy and sad music [75], suggesting that CI users detect a change from slow to fast tempo as accurately as the reverse (ie. from fast to slow).

Overall reaction time for the original condition was not significantly different between the two groups in the present study (3.8 ± 1.9 s CI users; 3.9 ± 1.9 s children with normal hearing; see Fig 3A), whereas our group has previously shown significantly slower responses for CI users [75]. Differences could be related to methodological and cohort differences between the present and previous studies. While responses to musical excerpts were collected by pushing a button in both studies the use of an iPad app versus an older button box may have offered a more interesting and compelling response paradigm than the previous method. However, if response method was to significantly alter behavior then similar decreases in response times would be expected in both normal hearing and implanted children and not specific to one group.

Age at test and length of electrical experience only slightly differed between the current and previous cohorts (~ 2 and 1 years respectively) and therefore likely did not significantly impact the differences noted in response times. On the other hand, unilateral auditory deprivation was limited (mean 2.5 years) in our current cohort of bilaterally implanted and bimodal users, whereas our previous cohort was unilaterally implanted and deaf in the contralateral ear. Though the MEI task does not evaluate binaural hearing, bilateral CI use has been shown to ease the increased listening effort demonstrated in unilateral CI use [98]. Greater spectral resolution can also ease effort in at least normal hearing individuals [99, 100], and 11 of our children retained residual hearing in the non-implanted ear. This greater access to acoustic hearing may play a role in response time, although there were no significant differences in reaction times across all CI users in the present cohort (see Fig 4, lowest panel). Therefore, limiting unilateral deprivation and listening with two devices may be the significant factors in normalizing effort required to judge emotion in unaltered musical excerpts.

Although reaction times to unaltered musical excerpts were similar between normal hearing children and bilateral CI and bimodal users, the change in response time with altered excerpts differed. Normal hearing children took significantly longer than children with CIs to judge the emotion of music with altered cues (Fig 3A). This difference in additional response times was clearest when both cues were changed; children with normal hearing spent an extra 1.9 ± 1.5 s to make their judgments whereas CI users spent only an extra 0.3 ± 0.6 s. Children with normal hearing recognize the alterations whereas the CI children may not be as sensitive to the changes. Perhaps when mode and tempo cues were changed together, the children with normal hearing struggled to assign emotion to the excerpts without these two major cues. However, if the CI children could not detect the changes to the musical excerpts to the same extent, there may have been less deliberation and effort in making decisions. Therefore, we hypothesized that when mode and tempo were changed together, the normal hearing children struggled without their cues, whereas CI children could not detect the changes to the same degree, and therefore, did not require additional effort to make the decision, as if they “gave up” trying to choose between the happy and sad judgment.

#### 2) Increased access to acoustic information reduces cochlear implant users’ need to use tempo cues for judging emotion in music

Opinion changes in the present groups of children using cochlear implants were not associated with bilateral auditory deprivation (defined as aided thresholds >40 dB HL from 0.5–4 kHz bilaterally) or unilateral deprivation (defined as no stimulation in one ear). Therefore, it appears that the length of deprivation did not affect simple emotion identification skills as it does speech perception outcomes [101]. On the other hand, the longer children used CI(s), the less they were affected by changes in mode cues and the more they relied on tempo. Not only did the electrical experience affect changes in opinion, but the total type of experience as well. Bimodal users who received a CI at older ages, and thus had longer access to acoustic sound in both ears, were less swayed by tempo changes than children who only had access to hearing through the electrical pulses of the CI because of poorer hearing thresholds (Fig 5A). By contrast, bilateral CI users showed no effect of total (mainly CI) time in sound on their use of tempo cues. Therefore, children with greater acoustic sound experience were less affected by tempo cues than those using bilateral CIs. Perhaps the complementary acoustic information prevented increased reliance on tempo by promoting the use of mode cues and/or reducing a developmental shift toward tempo cues. Furthermore, age at first implantation and age at test did not affect overall performance, which also supports the notion that hearing experience rather than absolute age is important for developing emotion identification in music. With this in mind, the ability of children using CIs to identify emotion in music did not strictly derive from their age, but from their type of hearing experience. It is difficult to isolate the separate contributions of pre- and post-implant acoustic experience as pre-implant experience was strongly correlated with both total time in sound (R = 0.63, p = 0.04) and post-implant experience (R = -0.80, p = 0.003) in our bimodal group, and there were very few children using only electrical hearing through bilateral CIs who had access to acoustic hearing for long periods prior to implantation. Nonetheless, our previous data on music perception in a cohort of unilateral CI users who did not use a hearing aid in the non-implanted ear suggest that acoustic experience pre-implant can benefit music perception with the CI [66]. Clearly, future studies should address etiology and characteristics of hearing loss, as well as duration of stimulation or access to the fine resolution in acoustic hearing on emotion perception in music.

If hearing experience is an important factor for judging emotion in music, it is surprising that no significant effect of musical training was found in pediatric CI users (Fig 4), even when accounting for total time in sound, CI experience and age at implantation (Fig 5). It is likely that no matter how much training is given to children with cochlear implants, they will not be able to perceive emotion in music normally (ie. using mode cues). The current research indicates that there remains technological constraints (ie. limited number of electrodes, poor dynamic range representation, coarse spectral resolution, speech-centered coding strategies), together with biological and acoustical peculiarities (ie. auditory nerve degeneration and abnormal auditory cortex activation) [76, 81, 102] that ultimately could explain why CI users will always be inclined to use their own adaptive strategy to detect emotions.

### C) Experiment 3: Mode cues must be distorted to impair processing of emotion in music

#### 1) Pink noise masks but does not distort mode cues in music

By introducing pink noise and degrading the signal through two vocoders, we purposely and successfully decreased the accuracy of individuals with normal hearing to that of unilaterally implanted CI children (Fig 6). Out of four different types of noise, only pink noise sufficiently decreased accuracy when tempo cues were altered. Yet, even though musical excerpts were barely audible in the presence of competing pink noise, participants were still able to correctly identify the emotions conveyed by the melodies to a similar extent as children with unilateral CIs in an easier quiet environment. Despite their accuracy, children with normal hearing required more time to assess the musical affect in pink noise than the vocoded music. They likely struggled to perform the task when their preferred mode cues were present but masked, rather than distorted by the vocoder, indicating that individuals with normal hearing attempt to use mode cues whenever possible.

In the vocoded conditions, accuracy in the normal hearing adults was similar to the unilateral CI group with similar reaction times, and their behavior mirrored CI users when cues were altered (Fig 7): opinions changed little with mode changes but changed considerably from the original with tempo changes. This was not the case when listening to music in pink noise, in which case opinion only changed when tempo cues were changed. Thus, effective use of temporal information by CI users is a compensatory strategy shared by normal hearing peers who do not have access to accurate pitch cues. Moreover, change in reaction time became similar between normal hearing listeners in these degraded conditions and the CI group in quiet. This means that the different use of cues by children using CIs is not fixed by having bilateral deafness, but suggests that children using more sophisticated CI devices delivering finer spectral information could potentially alter their processing strategies to depend more on mode than tempo cues. Indeed, the improved use of mode cues by bimodal users is in keeping with this idea (Fig 5).

#### 2) Mode distortion cannot be compensated by musical training

Musical experience accentuated the bias of using mode when assigning emotion to music. Participants with musical experience tried to use any available pitch information even when access to the signal declined with noise. Individuals with normal hearing and musical experience changed their opinion to a greater extent with mode changes than non-musicians, and this behavior was most evident when all available cues were present in the quiet condition. However, there was less effect of mode changes on musicians’behavior as pitch cues became distorted or masked (vocoder 32, pink noise, and vocoder 22 conditions, respectively). On the other hand, non-musicians responded similarly to mode changes across noise conditions, regardless of their age. Musicians’ opinions did not significantly change across age in pink noise, where presumably there were still some pitch cues, albeit masked to some extent. Though hearing in pink noise is a very challenging task, it is known that musicians have better speech-in-noise perception compared with non-musicians, even during pivotal childhood developmental years [103]. This is especially evident in binaural hearing conditions, as binaural processing is an important mechanism that promotes hearing in complex listening environments [104]. Interestingly, music experience did not appear to affect reliance on tempo, even with a pitch-deprived signal. This confirms that formal training can enhance interval discrimination abilities [95], but may have little effect on tempo reliance in emotion identification tasks. Moreover, we observed a trend that reaction times lengthened to a greater extent with age when individuals with normal hearing listened to the mode-altered musical excerpts, reflecting the increased task difficulty for older individuals who became increasing hardwired to relying on pitch for emotion judgments. When pitch cues were present, though changed in tonality, people with normal hearing and musical experience changed their opinion progressively with training (Fig 8). Interestingly, reducing or degrading pitch access reduced the difference until it disappeared in those conditions where pitch information was the most deprived; in this scenario (i.e., vocoder 22), length of musical training had no bearing on opinion changes with mode changes. A similar trend could be seen when tempo changed, except that the effect was immediate once the noise or filtering occurred.

### D) The unique role of pitch in perception: implications for CI users

Findings from the present study confirm that there is an increasing use of mode cues to judge emotion in western music in normal development. This strong dependency reflects the unique use of pitch in the context of music perception relative to speech perception [105]. Indeed, the brain develops specialized processing for speech which is different from music [106]. Networks involved in temporal coding and speech processing are lateralized to the left cortex whereas right cortical activity is stronger in response spectral cues in music [5, 6]. The increased sensitivity to pitch in music as compared to speech perception is consistent with our findings that individuals with normal hearing could no longer use mode cues when the music was vocoded to 22 channels and had to switch to tempo cues to detect emotion in music. By contrast, speech perception remains normal even in noise with 22 channels and only begins to deteriorate when far fewer channels of vocoded speech are available [107].

The strong dependence of pitch for emotion perception in music makes the MEI an excellent tool to study effects of poor access to pitch cues. It is clear that neither unilateral [58] nor bilateral CIs provide children with sufficient access to pitch to use mode cues to judge emotion in music. The importance of access to even low frequency pre-implant residual hearing in development was shown to be helpful for musical hearing post-implant [66]. In the present study, children with some access to acoustic pitch cues through hearing aid use in the ear opposite to the CI, showed a reduced reliance on tempo cues. This indicated that they retained better use of pitch cues than their implanted peers. Future studies can use the MEI test to access the association between degree of residual hearing and ability to access mode (ie pitch cues). At present, it is clear that children’s access to pitch cues should be preserved wherever possible. Efforts to preserve residual hearing include bimodal fittings, as in the group studied here, soft implantation surgery [108], and changes to electrode design [109]. For children with little residual hearing, improvements to the implant technology are needed to better represent the fine temporal structure of music and speech. Possible strategies include increasing the number of available electrodes [110], providing focused stimulation [111], and/or implementing new coding strategies [112]. The lack of access to pitch in the CI may suggest that music training in CI users focus on other potential benefits such as motor learning [113] and enjoyment of music [114]. Indeed, many children with CIs love to engage with music by listening, singing, dancing, and playing an instrument [115].

## Conclusion

The combined findings of the 3 experiments suggest that with normal hearing, humans in western culture develop a predisposition toward mode cues when asked to judge simple emotions in music. This development is altered when access to pitch cues is deprived by deafness and cochlear implant use. The new cohorts of bilateral cochlear implant users and bimodal users indicate that improving access to acoustic hearing reduces the abnormal switch to tempo to judge emotion in music. The importance of access is highlighted by the immediate switch by individuals with normal hearing to use cochlear implant-like strategies of tempo upon acute degradation of pitch cues. This means that, for individuals with hearing loss, pitch cues must be preserved (for those with residual hearing) and/or replaced by improvements to cochlear implant technology.

## Supporting Information

S1 Dataset (XLSX)
